# Alcohol Use Disorder in Southwestern and Northern Uganda: Prevalence and associated factors

**DOI:** 10.1371/journal.pgph.0005785

**Published:** 2026-01-02

**Authors:** Abraham Muhwezi, Tom Murungi, Pius Musinguzi, Davis Akampumuza, Mary Samantha, Moses Ocan, Henry Ochola, Godfrey Zari Rukundo, Samuel Maling, Edith K. Wakida, Celestino Obua

**Affiliations:** 1 Department of Community Health, Mbarara University of Science and Technology, Mbarara, Uganda; 2 Department of Midwifery, Lira University, Lira, Uganda; 3 Department of Nursing, Mbarara University of Science and Technology, Mbarara, Uganda; 4 Department of Governance and Planning, Mbarara University of Science and Technology, Mbarara, Uganda; 5 Department of Pharmacology and Therapeutics, Makerere University, Kampala, Uganda; 6 Department of Obstetrics and Gynaecology, Mbarara University of Science and Technology, Mbarara, Uganda; 7 Department of Psychiatry and Behavioral Neurosciences, McMaster University, Hamilton, Ontario, Canada; 8 Department of Psychiatry, Mbarara University of Science and Technology, Mbarara, Uganda; 9 Department of Medical Education and Center for Brain and Behavioral Health, California University of Science and Medicine, Colton, California, United States of America; 10 Department of Research and Development, Alpha Centre for Research Administration, Mbarara, Uganda; 11 Vice Chancellor’s Office, Mbarara University of Science and Technology, Mbarara, Uganda; London School of Hygiene and Tropical Medicine Faculty of Epidemiology and Population Health, UNITED KINGDOM OF GREAT BRITAIN AND NORTHERN IRELAND

## Abstract

Alcohol use disorder (AUD) among adults is a major public health concern globally. Alcohol use disorder affects livelihood and contributes to adverse health outcomes. We investigated the prevalence of AUD and associated factors among adults (≥ 18years) in Lira and Isingiro districts in Uganda. This was a cross-sectional study conducted among adults (≥18 years) in households, selected using multistage sampling. Data were collected using an interviewer-administered questionnaire and the Alcohol Use Disorder Identification Test (AUDIT) tool. Data were downloaded from the Kobo Toolbox interface into Microsoft Excel and then exported to STATA version 17 for cleaning and analysis. A total AUDIT score of eight or more was used to define AUD. Binary Logistic regression was used to analyze factors independently associated with AUD at a 95% confidence level. A total of 577 participants were recruited in the study, with a median age of 35 years (IQR: 26–46). Half, 50.8% (293/577) of the participants were male. Over a third, 39.9% (230/577) of the participants had AUD. Factors significantly associated with AUD were; being male (aOR = 8.01; 95% CI: 5.02–12.76), age group of 31–50 years (aOR = 2.00; 95% CI: 1.21–3.33), secondary level education (aOR = 0.63; 95% CI: 0.21–0.78), having family members who use alcohol (aOR = 4.09; 95% CI: 2.54–6.59), use of other substances (aOR = 1.79; 95% CI: 1.10–2.92), and presence of major stressors (aOR = 3.58; 95% CI: 2.22–5.78). Alcohol use disorder was highly prevalent among adults in Uganda. Being male, age, use of other substances of abuse, and alcohol use among family members were significantly associated with AUD. The Ministry of Health and other key stakeholders should prioritize AUD, strengthen the regulation of alcohol use in the country, and provide rehabilitation for individuals with AUD.

## Introduction

Alcohol use disorder (AUD) is a major global health concern and is associated with negative physiological and psychological health outcomes [1]. Worldwide, about 400 million adults suffer from AUD, and alcohol causes about 2.6 million deaths annually [2,3]. Additionally, about 52.2 deaths per 100,000 occur in the WHO African region due to alcohol [2]. Uganda has a high burden of alcohol consumption, with each person consuming, on average, twelve litres of alcohol annually [4,5]. Moreover, Alcohol use disorder affects nearly 10% of the adult population in Uganda [6] and contributes to 6.5% of all deaths nationally [7]. Particularly, higher alcohol consumption rates in adults are found in Northern (23.2%) and Western Uganda (21.4%) [6].

The harmful effects of AUD extend beyond mortality. Chronic alcohol use increases the risk of non-communicable diseases such as liver cirrhosis, cardiovascular disease, and cancers, while also weakening immunity and exacerbating infectious diseases like tuberculosis and HIV [8]. Psychologically, AUD is strongly associated with depression, anxiety, and other psychiatric disorders [8,9], and it contributes to interpersonal violence, road traffic injuries, and reduced work productivity (Lee et al., 2025). Collectively, these impacts place a heavy burden on households, communities, and national health systems [8,10].

Reported prevalence estimates of alcohol use disorder (AUD) vary considerably across different settings and populations. Studies have documented rates of 26.3% in the United States [11], 34.9% in Sub-Saharan Africa [12], 61.8% in Ethiopia [13] and 28.2% in Tanzania [14]. Ugandan studies have reported AUD prevalence rates ranging from 9 to 40% among the population [6,15,16]. Significantly, factors like male gender, education, older age, and marital status have been consistently associated with AUD among adult individuals [15–19]. However, evidence on the burden and determinants of AUD among adults in Uganda remains limited and fragmented, highlighting the need for further community-based studies.

Despite existing regulatory frameworks, such as the Enguli Act of 1966 and WHO’s SAFER initiatives, alcohol remains widely accessible due to weak policy enforcement and informal local production in Uganda [5,20–22]. Thus, nearly one in four adults experiences a mental illness [23], conditions that often co-occur with and are worsened by AUD [24]. Yet, evidence on the prevalence and determinants of AUD among adults in Uganda remains limited, with most existing studies focusing on adolescents in selected regions [15,16]. With the increasing burden of mental illnesses reported in Lira [25] and Isingiro districts [26], this study aimed to estimate the prevalence of AUD and associated factors among adult residents of Lira and Isingiro Districts.

## Methods

### Ethics statement

The protocol was reviewed and approved by the Gulu University Research Ethics Committee (GUREC-2024–984). Administrative clearance was obtained from Lira and Isingiro districts before field data collection. All participants provided written informed consent. Participation was voluntary, with the right to withdraw anytime. We ensured participants’ privacy, confidentiality, and anonymity by conducting interviews in a private setting and de-identifying data. Identifiers were accessible only to the research team for quality checks. Electronic data were password-protected, and physical forms were kept locked. This study complied with all relevant ethical guidelines for human subjects’ research.

### Study design and setting

This was a community-based cross-sectional study conducted among adult residents in households in Lira and Isingiro districts in northern and southwestern Uganda. Lira and Isingiro districts are located 337 km north and 303 km southwest of the capital Kampala, respectively. The study was conducted between January and February 2025. A national survey reported a high burden of alcohol consumption in northern (23.2%) and western (21.4%) regions of Uganda [6].

### Study population, sample size and sampling

The study enrolled adult (≥18 years) participants in households and excluded pregnant women and individuals with cognitive impairments or those who were severely ill at the time of data collection. Pregnant women were excluded from the study because pregnancy-related physiological changes and discomforts, such as fatigue, nausea, and vomiting, could interfere with participants’ ability to reliably complete interviews. Individuals with cognitive impairments or severe illnesses were excluded because such conditions may limit participants’ ability to provide informed consent and reliably respond to questions, thereby compromising both data quality and ethical standards. Lira and Isingiro districts were purposively selected to represent the Northern and Southwestern regions of Uganda, where prior surveys reported higher alcohol consumption [6].

A sample size of 684 was calculated using the Kish–Leslie formula (1965), assuming a prevalence of 50%, a design effect of 1.5, a 10% non-response rate, and a 95% confidence level. At the national level, Uganda has 146 districts; of these, two (Lira and Isingiro) were purposively chosen. After this purposive first stage, the study employed multistage probability sampling for all subsequent stages. At the time of the survey, Lira District had 13 sub-counties, while Isingiro had 14. Each sub-county contained approximately 40–70 villages. In the first probability-based stage, three sub-counties were randomly selected from each district. In the second stage, six villages per selected sub-county were chosen using probability proportional to size (PPS), based on the 2024 Uganda Bureau of Statistics (UBOS) census village population counts, yielding 36 villages in total. PPS was applied only to determine which villages were selected, such that larger villages had a higher probability of inclusion. In the third stage, we sampled a fixed number of 19 households from every selected village, for a planned total of 684 households. This constant number was adopted for operational feasibility during fieldwork. Because villages were selected using PPS while the number of households sampled per village was fixed, the probability of household selection differed across villages, resulting in unequal selection probabilities. We explicitly acknowledge this feature of the design. Within each household, one eligible adult was randomly selected.

We targeted 684 households, but only 577 individuals participated, due to refusal or absence at the time of the survey. The assumed design effect of 1.5 (for cluster size m = 19) corresponds to an inter-class correlation coefficient of approximately 0.028, which suggests that clustering effects were likely modest but unaccounted for in our analysis. Using the 2024 Ugandan population census data for Lira and Isingiro districts, we proportionately recruited 266 participants from Lira district and 311 participants from Isingiro district [27,28].

### Data collection procedures

Interview data were collected using a researcher-administered questionnaire. The questionnaire items were developed from previously published studies [15,29,30]. In addition, the questionnaire incorporated the ten standard AUDIT (Alcohol Use Disorder Identification Test) questions [31]. The questionnaire was pretested among adult individuals in Mbarara city, and the findings were used to adjust the tool. The questionnaire collected data on the following areas: (i) socio-demographic characteristics, (ii) Alcohol use disorder and (iii) factors associated with alcohol use disorder. The 10-item Alcohol Use Disorders Identification Test (AUDIT) [32] evaluates three main areas: (1) Alcohol Consumption, with questions related to the frequency and quantity of alcohol intake, (2) Alcohol-related problems, and (3) Dependence Symptoms. The AUDIT was scored from 0 to 40, with a total of 0 indicating non-alcohol consumers, 0–7 indicating low-risk consumption, 8–15 indicating hazardous or harmful drinking, 16–19 indicating moderate AUD and 20–40 indicating severe AUD or alcohol dependence [33]. This study defined Alcohol Use Disorder (AUD) as a total AUDIT score of eight or higher, and the same classification has been used by similar previous studies [13,18,34]. Major stressors in this study referred to challenges experienced by individuals across economic (e.g., poverty, financial insecurity), social (e.g., family conflict, community disruption, stigma), and psychological (e.g., trauma and emotional distress) domains.

The interviews were done by six research assistants who had a Bachelor of Science degree in nursing. Before the field data collection, the research assistants were trained on the study protocol and the ethical conduct of research. Research assistants approached and recruited potential study participants from households. In each household, participants were screened for eligibility, and where more than one eligible adult was found, simple random sampling was used to identify one individual to be recruited into the study. Written informed consent was then obtained from the study participants before the interview. Data were collected using the questionnaire built into the Kobo Collect Android software. At the end of each data collection day, the research assistants reviewed the entries for completeness, and the records were synchronized to the main server (https://kc.humanitarianresponse.info/).

### Data management and analysis

The data collection interface, based on a questionnaire, was developed using the Kobo Collect software with built-in checks. The data set was downloaded into a Microsoft Excel spreadsheet, checked for completeness, and exported to STATA version 17 (STATA Corp, Texas, USA) for analysis. Continuous variables were analyzed using descriptive statistics (frequencies, proportions, means, and standard deviations). Categorical variables were summarized using proportions. Factors associated with alcohol use disorder were analyzed using multivariable logistic regression. The model was built using a backward conditional approach, with all variables having p-values < 0.2 at the bivariate level included in the model. Model diagnostics were performed to assess robustness and fit. Multicollinearity was evaluated using variance inflation factors (VIFs). Model specification was examined using the link test, and goodness of fit was assessed with the Hosmer–Lemeshow test. All analyses were conducted at a 95% confidence level, and adjusted odds ratios (aORs) with 95% confidence intervals are presented.

Although our sample size estimation incorporated a design effect of 1.5 to account for clustering at the village level, the final analysis did not apply survey weights. This was due to the mixed sampling structure, purposive selection at the district level followed by probability sampling at lower levels, as well as incomplete sampling frame information needed to accurately compute household-level weights. Consequently, the logistic regression models were conducted as unweighted analyses.

## Results

### Participants’ characteristics

A total of 577 study participants were recruited, with half, 50.8% (293/577), being male. The median age of study participants was 35 years (IQR: 26–46). The majority, 70.0% (404/577), had no formal education, and 25% (144/577) of the respondents reported current use of other substances, including marijuana, khat, shisha and Tobacco*.* Most, 80.9% (409/577) of the participants reported having friends who use alcohol (Table 1).

**Table 1:** Participant characteristics of adult residents of Lira and Isingiro Districts, Uganda, collected in Jan and Feb 2025, (N = 577).

Characteristic	Description	Frequency (n)	Percentage (%)
**Age categories (years)**	18-30	236	40.8
	31-50	231	40.1
	51+	110	19.1
**Sex**	Female	284	49.2
	Male	293	50.8
**level of education**	None/primary	404	70.0
	Secondary	100	17.3
	Tertiary	73	12.7
**Employment**	Employed	505	87.5
	un employed	72	12.5
**Marital status**	Single	148	25.7
	co-habiting	94	16.2
	Married	335	58.1
	Yes	133	23.1
**Use of other substances (**marijuana, khat, shisha and Tobacco)	No	433	75.0
	Yes	144	25.0
**Think alcohol is harmful**	Yes	514	89.1
	No	41	7.1
	Unsure	22	3.8
**Having friends who use alcohol**	No	110	19.1
	Yes	467	80.9
**presence of major stressors**	No	193	33.5
	Yes	384	66.5

Other substances were marijuana, khat, shisha and Tobacco. Major stressors were: job loss, terminal illness, financial losses and loss of a loved one.

### Prevalence of Alcohol Use Disorder among adult residents of Lira and Isingiro Districts

The overall prevalence of Alcohol Use Disorder among adult residents was 39.9% (230/577; 95% CI: 35.8%–44.0%). Of these, 74.8% (172/230) were males and 25.2% (58/230) were females. The prevalence of AUD was higher among study participants in Isingiro district, with 55.2% (127/230), compared to 44.8% (103/230) in Lira district.

Among the participants, 60.1% (347/577) were classified as low-risk drinkers/had no AUD (AUDIT <8), 17.5% (101/577) were identified as hazardous drinkers, 7.5% (43/577) were categorized as having moderate AUD, and 14.9% (86/577) met the criteria for alcohol dependence.

### Factors associated with Alcohol Use Disorder among adult residents of Isingiro and Lira Districts, Uganda

From bivariate analysis, the factors which were significantly associated with AUD included age group 31–50 years (*p-value*, 0.003), male gender (*p-value*, 0.001), secondary level education (*p-value,* 0.001), employment (*p-value,* 0.001), having family members using alcohol (*p-value*, 0.001), thinking alcohol is harmful (*p-value,* 0.031), having a diagnosis of mental illness (*p-value*, 0.023), substance use (*p-value*, 0.001), and presence of major stressors (*p-value,* 0.001).

Factors which were associated with AUD with a p-value <0.2 at bivariate analysis were considered for multivariable analysis. A multivariable logistic regression was used to establish the factors associated with AUD. Model fitness and assessment of how well the predicted probabilities aligned with the observed outcomes were tested using the Hosmer and Lemeshow test, which gave a p-value of 0.63, indicating that the model fit the data. From the multivariable logistic regression analysis, being male (aOR = 8.01; 95% CI: 5.02–12.76; *p* < 0.001), age group 31–50 years (aOR = 2.00; 95% CI: 1.21–3.33; *p* = 0.007), having attained secondary school education (aOR = 0.63; 95% CI: 0.21–0.78; *p* = 0.001), having family members who use of alcohol(aOR = 4.09; 95% CI: 2.54–6.59; *p* < 0.001), use of other substances (aOR = 1.79; 95% CI: 1.10–2.92; *p* = 0.019), and presence of major stressors (aOR = 3.58; 95% CI: 2.22–5.78; *p* < 0.001) were significantly associated with AUD (Table 2).

**Table 2:** Factors associated with Alcohol Use Disorder Among Adult Residents of Lira and Isingiro Districts at bivariate and multivariable logistic regression.

Characteristic	Description	cOR, 95% CI	p-value	aOR, 95% CI	*p-value*
**Age**	18-30 years	Ref		Ref	
	31-50 years	1.80 (1.24- 2.24)	0.002	2.00 (1.21- 3.33)	**0.007**
	51 + years	1.50(0.94- 2.40)	0.087	1.25 (0.67-2.31)	0.485
**Sex**	Female	Ref		Ref	
	Male	5.53(3.82-8.02)	**< 0.001**	8.01(5.02-12.76)	**< 0.001**
**Education level**	None/primary	Ref		Ref	
	Secondary	0.43 (0.26- 0.72)	**0.001**	0.63 (0.20- 0.67)	**0.001**
	Tertiary	1.33 (0.81- 2.21)	0.252	1.08 (0.58-2.03)	0.816
**Having family members who use alcohol**	No	Ref		Ref	
	Yes	2.09 (1.47-2.99)	**< 0.001**	4.09 (2.54-6.59)	**< 0.001**
	Don’t know	1.65 (0.81- 3.39	0.169	2.20 (0.91-5.33)	0.081
**Think alcohol is harmful.**	Yes	Ref		Ref	
	No	2.02 (1.06- 3.84)	**0.031**	1.72 (0.77-3.84)	0.183
	Unsure	0.90 (0.37- 2.20)	0.824	1.69 (0.53-5.39)	0.374
**Use of other substances**	No	Ref		Ref	
	Yes	2.64 (1.80- 3.89)	**< 0.001**	1.79 (1.10-2.92)	**0.019**
**Presence of a major stressor**	No	Ref		Ref	
	Yes	3.31 (2.23- 4.91)	**< 0.001**	3.58 (2.22-5.78)	**< 0.001**

AOR-adjusted odds ratio, COR-crude Odds Ratio, CI-Confidence Interval. Other substances were marijuana, khat, shisha and Tobacco. Major stressors were: job loss, terminal illness, financial losses and loss of a loved one. Employment status was excluded from the multivariable model because it showed evidence of collinearity with other socioeconomic variables, particularly education level and presence of major stressors, which led to model instability when included simultaneously.

## Discussion

Over a third of the study participants had alcohol use disorder, with a higher prevalence among males. The factors significantly associated with AUD include use of other substances, age group 31–50 years, presence of major stressors, and having a family member who uses alcohol.

Almost 40% of the participants in this study had alcohol use disorder. This finding differs from that of a previous study, which reported an AUD prevalence of 9.8% among adult individuals in Uganda [6]. In addition, the findings of this study are higher than the 33.3% AUD prevalence reported in a systematic review of studies in Sub-Saharan Africa [35]. However, our finding is similar to other studies conducted in Eastern and Central Uganda [9,15,36]. This higher prevalence of AUD in Uganda can be attributed to the easy accessibility and affordability of alcohol in most parts of the country [15,22]. Likewise, the high prevalence of AUD may be attributed to several factors, including pervasive and indiscriminate alcohol advertising, as well as weak regulatory frameworks that fail to effectively control the production and distribution of alcoholic beverages [36]. This prevalence is high despite the implementation of alcohol control measures like the banning of sachet packaged alcohol and the existence of policies like the Enguli Act of 1966 [20] and the alcohol control policy in Uganda [21]. This, however, is a result of weak and uncoordinated enforcement of laws and policies on alcohol, due to a lack of sufficient human and financial resources to enforce the law [37,38]. Additionally, the penalties specified by these policies are outdated in the current context and are too light to safeguard the population against harmful alcohol use [37,39]. Hence, this highlights the urgent need for the Ugandan government to revise and strengthen existing policies to address the informal production and sale of alcohol [37,40]. Additionally, it is important to regulate the marketing of alcoholic beverages [41] and to incorporate alcohol harm education interventions into local health and community programs to regulate alcohol abuse and alcohol use disorder [42,43]. Future research should explore factors contributing to Uganda’s high AUD prevalence, including informal alcohol production, accessibility, marketing, and regulatory gaps. Comparative studies could clarify how sociocultural and economic contexts influence drinking patterns. Intervention studies are needed to evaluate policy, taxation, and community-based programs to reduce alcohol misuse and AUD prevalence [44–46].

We found that being a male was significantly associated with AUD. Moreover, males had a higher AUD prevalence in our findings. This result is consistent with many other findings from both developed and developing countries [13,15,16,36,47]. The observation that male individuals consume alcohol more than females is in line with the traditional view that drinking is more tolerated among males [48]. Similarly, findings from a Ugandan study reported that alcohol drinking is a masculine adult activity with traditionally low rates among women in Africa and that alcohol is socially acceptable among men [22]. This underscores the need for gender specific interventions that challenge wrong myths that lead to alcohol and promote healthier coping strategies to help reduce AUD among men [49]. Future studies should employ longitudinal and analytical designs to clarify the cultural, social, and biological mechanisms underlying men’s increased likelihood of AUD. In addition, qualitative and mixed methods research is needed to examine how gender norms, masculinity ideologies, and social expectations shape alcohol use in this setting [50,51].

In this study, participants who reported having major stressors like chronic illnesses, stress, depression, and financial hardships had 3.58 times higher odds of AUD compared to those who had no major stressors. This concurs with findings from previous studies in Ethiopia [52], Australia [53] and Uganda [54]. A plausible explanation is that individuals may resort to alcohol as a coping mechanism to manage stress or emotional pain [55]. However, while alcohol might offer temporary relief, relying on it as a coping strategy is problematic. It may mask underlying mental health conditions, delay individuals from seeking appropriate psychiatric or psychological support, and potentially worsen the severity of the psychological condition. This highlights a critical public health concern: the use of alcohol to cope with psychosocial stressors may reinforce a harmful cycle that increases vulnerability to AUD and complicates recovery. These findings highlight the importance of early mental health screening and integrated interventions that focus on emotional well-being [56]. To achieve this, the Ministry of Health should incorporate mental health screening into routine primary care and community outreach programs and collaborate with district health offices to establish psychosocial support services. Furthermore, the Uganda Alcohol Policy Alliance (UAPA) should advocate for policies that enhance mental health literacy and increase access to early intervention and community-based mental health services, especially in areas with high AUD prevalence.

Our findings revealed that adults aged 31–50 years had 2.0 times higher odds of AUD compared to those aged 18–30 years. Similarly, a study in Eastern Uganda found that alcohol use was least among individuals aged 18–29 years, and highest among those aged 45–50 years [15]. The lower prevalence of AUDIT positive scores in younger individuals may stem from social desirability bias in self-reports, leading them to downplay their drinking habits or related issues. It is also evident that younger men are often non-respondents in alcohol studies and tend to under-report their alcohol consumption [57]. Likewise, a previous cohort study revealed that AUD symptoms reached the peak around the age of 35–45 years, after which the prevalence started to decrease [58]. The age onset of alcohol related problems is a key determinant of AUD severity and treatment outcome [59]. For instance, middle-aged adults with AUD were found to have increased risks of hypertension, pulmonary diseases, cerebrovascular diseases, malnutrition, metabolic disorders and cancer than younger and older adults with AUD [60]. This age group, often characterized by heightened responsibilities such as career demands, family obligations, and financial pressures, may be particularly vulnerable to alcohol misuse as a form of coping. Such age-related variation in the prevalence and severity of AUD necessitates targeted and age-specific interventions to avert AUD in the general population. Comparative studies across different age groups would help clarify how age-specific social roles and health risks shape patterns of alcohol use. In addition, intervention trials should evaluate the effectiveness of prevention and treatment strategies tailored to the needs of distinct age groups. Research is also needed to examine how occupational stress, family obligations, and financial pressures contribute to alcohol misuse among middle-aged adults [61,62].

Participants who used other substances like marijuana, khat, shisha and Tobacco had 1.79 times higher odds of AUD compared to non-users. Previous studies revealed cigarette smoking [63], chewing khat [13], and concomitant use of non-alcoholic substances [64] and other illicit drugs [47] to be associated with AUD. These results suggest that there is a general tendency towards addictive behaviours where individuals who use one drug are likely to abuse other drugs. Alcohol and other drugs increase the release of dopamine, which is responsible for reinforcement and pleasure [11]. This highlights the need for national policies to adopt integrated substance use prevention and treatment strategies that do not address alcohol in isolation, but rather target multiple substances concurrently through community-based and primary healthcare interventions [65]. Future research should employ longitudinal and analytical study designs to clarify the causal pathways underlying polysubstance use, while also examining context-specific risk factors such as cultural practices, mental health comorbidities, and socioeconomic influences [66]. Furthermore, interventional studies are needed to evaluate the effectiveness of integrated prevention and treatment approaches that address alcohol and non-alcohol substance use concurrently [67].

The study also found that participants who had family members who used alcohol had 4.09 times higher odds of AUD compared to those whose family members didn’t. This finding aligns closely with social learning theory, which posits that individuals model behaviours observed in significant others, especially when those behaviours appear to be rewarded or go unpunished [68]. Similarly, studies in Uganda revealed that parental and sibling alcohol use were associated with AUD among adolescents [69,70]. In addition, other studies from Ethiopia support the claim that having a family member who uses alcohol is positively associated with AUD [71]. Our findings suggest that familial modelling both accelerates initiation and entrenches maladaptive drinking trajectories. Thus, programs that engage entire households, such as educating parents about their drinking, improving family communication, and promoting alternative activities, may more effectively disrupt the intergenerational transmission of AUD risk. We recommend future research to examine the mechanisms through which familial alcohol use influences AUD, including genetic, environmental, and social learning pathways. Longitudinal and analytical studies could clarify how parental and sibling drinking behaviours affect initiation, escalation, and persistence of alcohol use across generations. Comparative studies across different family structures and cultural contexts would help identify which familial and environmental factors most strongly contribute to AUD. Additionally, intervention studies are needed to evaluate the effectiveness of family-based programs that target parental drinking, enhance family communication, and promote alternative coping strategies to reduce intergenerational transmission of AUD risk.

This study provides evidence on the prevalence and associated factors of AUD among adult residents in rural Uganda, laying a foundation for targeted, evidence-based public health interventions and informed alcohol policy development. A key methodological strength of this study is its use of a community-based sampling approach, which enhances the generalizability of the findings to the rural adult population. Additionally, the study employed the WHO-validated AUDIT tool, ensuring a reliable and standardized assessment of alcohol use patterns. The inclusion of diverse sociodemographic variables also allowed for a comprehensive analysis of potential risk factors, and lastly, the study had a good response rate, 84%.

This study has some limitations that may affect the interpretation of the findings. Reliance on self-reported data may have introduced reporting bias, potentially influencing accuracy. In addition, the cross-sectional design limits causal inference between the identified factors and AUD. Third, although the sample size calculation incorporated a design effect of 1.5, the final analysis did not apply survey weights. Fourth, because villages were selected using PPS while a fixed number of households was sampled in each village, the resulting selection probabilities were unequal. The use of unweighted data may therefore affect variance estimation and lead to confidence intervals that are narrower than they would be under fully weighted survey analysis. Fifth, although 684 households were targeted, only 577 individuals ultimately participated, raising the possibility of non-response bias. Individuals who were absent or declined participation may differ in alcohol use patterns from those who were interviewed, which could influence prevalence estimates. Future longitudinal studies are warranted to establish temporal relationships and clarify causal pathways.

## Conclusion

The prevalence of alcohol use disorder was high among adults, with males disproportionately affected. AUD was significantly associated with several factors, including age, use of other substances, major life stressors, level of education, and alcohol use by a family member. Based on these findings, the Uganda Alcohol Policy Alliance (UAPA), in collaboration with the Ministry of Health and local government health departments, should promote family-centred prevention strategies and integrate targeted alcohol harm reduction messages into existing public health campaigns. The Government should also strengthen enforcement of alcohol control laws, such as regulating sales hours and marketing, while establishing structured peer support and counselling services at the community level to reduce alcohol dependence and address associated risk factors.

## Supporting information

S1 Data (XLSX)

S2 Data (DOCX)
