# Supplementary material for: Alcohol Use Disorder in Southwestern and Northern Uganda: Prevalence and associated factors
Source: PLOS Glob Public Health. 2026 Jan 2;6(1):e0005785. doi: 10.1371/journal.pgph.0005785 (PMC12758784; doi:10.1371/journal.pgph.0005785)
Supplement: S2 Data — (DOCX) [file pgph.0005785.s002.docx]

**PART I: AUDIT**

Section A

Thank you for participating in this study. This interview aims to understand the prevalence of AUD in selected Rural districts of Uganda

Date of Interview: …………………… Venue of Interview: …………………… Interview starting time: ………… Interview ending time (10-15) minutes

Participant ID: .................................

**Demographic information**

1. Age (years)…………………….
2. Sex ………………………….
3. Level of education/ qualification ……………………
4. Role (employment) ..........…
5. What is your monthly income level?
6. Less than 200,000 UGX
7. 200,000 - 500,000 UGX
8. Above 500,000 UGX
9. What is your relationship status?
10. Single
11. In a relationship
12. Married

**Please circle the answer that is correct for you**

1. How often do you have a drink containing alcohol?
2. Never
3. Monthly or less
4. 2-4 times a month
5. 2-3 times a week
6. 4 or more times a week
7. How many standard drinks containing alcohol do you have on a typical day when drinking?
8. 1 or 2
9. 3 or 4
10. 5 or 6
11. 7 to 9
12. 10 or more
13. How often do you have six or more drinks on one occasion?
14. Never
15. Less than monthly
16. Monthly
17. Weekly
18. Daily or almost daily
19. During the past year, how often have you found that you were not able to stop drinking once you had started?
20. Never
21. Less than monthly
22. Monthly
23. Weekly
24. Daily or almost daily
25. During the past year, how often have you failed to do what was normally expected of you because of drinking?
26. Never
27. Less than monthly
28. Monthly
29. Weekly
30. Daily or almost daily
31. During the past year, how often have you needed a drink in the morning to get yourself going after a heavy drinking session?
32. Never
33. Less than monthly
34. Monthly
35. Weekly
36. Daily or almost daily
37. During the past year, how often have you had a feeling of guilt or remorse after drinking?
38. Never
39. Less than monthly
40. Monthly
41. Weekly
42. Daily or almost daily
43. During the past year, have you been unable to remember what happened the night before because you had been drinking?
44. Never
45. Less than monthly
46. Monthly
47. Weekly
48. Daily or almost daily
49. Have you or someone else been injured as a result of your drinking?

0.No

2.Yes, but not in the past year

4.Yes, during the past year

1. Has a relative or friend, doctor or other health worker been concerned about your drinking or suggested you cut down?
2. No

2.Yes, but not in the past year

4. Yes, during the past year

**PART II: Factors associated with Alcohol Use Disorder**

1. Have you experienced any major stressors in your life (e.g., job loss, relationship issues, financial difficulties)?
   1. No
   2. Yes
2. Do you tend to drink alcohol to cope with stress or negative emotions?
   1. No
   2. Yes
3. Do you have a family history of alcohol use disorder (AUD) or other substance use disorders?
   1. No
   2. Yes
   3. Don’t know
4. Do you feel pressured to drink in social situations?
   1. No
   2. Yes
5. Do you have a support network (family, friends, professional help) to turn to when facing difficulties?
6. No
7. Yes
8. How harmful do you think excessive alcohol consumption is for your health?
9. Extremely harmful
10. Somewhat harmful
11. Not harmful
12. Unsure
13. Have you ever been diagnosed with a mental health condition (e.g., depression, anxiety, PTSD)?
14. No
15. Yes
16. Which of the following substances do you use/take? Tick all that applies.

| **Substance** | **No** | **Yes** |
| --- | --- | --- |
| Cigarette smoking |  |  |
| Chewing Khat |  |  |
| Marijuana use |  |  |
| Shisha |  |  |
| Others |  |  |

1. Have you ever been diagnosed with a chronic illness?
2. No
3. Yes
4. If in a relationship/married, does your partner engage in heavy alcohol use?
5. No
6. Yes
7. Do you have a family member that drinks alcohol?
   1. No
   2. Yes
8. Have your friends or peers ever encouraged you to drink more alcohol?
9. No
10. Yes
11. In your community, is alcohol consumption considered a normal or important part of social gatherings?
12. No
13. Yes
